# Comparative analysis of postoperative outcomes in men and women following total hip replacement surgery

**DOI:** 10.1186/s13018-026-06811-6

**Published:** 2026-04-03

**Authors:** Sandra Ruta, Til Leander Riedl, Patrick Orth, Christian Götze, Filippo Migliorini, Julian Koettnitz

**Affiliations:** 1 Ruhr University Bochum, Bochum, Germany; 2 Ruhr-University Bochum (RUB), Medical Faculty,, University Hospital Auguste-Viktoria, Department of General Orthopedics, Bad Oeynhausen, Germany; 3 Department of Orthopaedics and Trauma Surgery, RWTH Aachen University, Aachen, Germany

**Keywords:** Harris hip score, Satisfaction, sex, Total hip arthroplasty, Quality of life

## Abstract

**Background:**

Total hip arthroplasty (THA) is a widely performed procedure to alleviate pain and restore function in patients with advanced hip osteoarthritis. Despite extensive research, the question of sex disparities remains inconclusive, with studies showing comparable or different clinical outcomes.

**Methods:**

A prospective analysis was conducted on 167 patients who underwent total hip arthroplasty (THA) between 06/2022 and 06/2023 at a university hospital to analyses sex-specific outcomes. Demographic data, the mobility, the use of walking aids, pre- and postoperative range of motion, pain and the HHS (Harris Hip Score) after six months were collected and analysed. Data analyses were conducted with SPSS Version 29.0.

**Results:**

The mean age of the patients was 66.9 ± 10.5 with a percentage of women (w) of 60.5%. After six months the range of motion between the sexes was significantly different with more motion deficits for men (m). For example, women revealed a significantly greater total range of motion (ranks: 70.8 vs. 92.6; *p* = 0.001) with a higher degree (°) of hip flexion ((w) 103.0° ± 7.5° vs. (m) 98.2° ± 7.4°; *p* = 0.001) and internal rotation ((w)13.3° ± 6.8° vs. (m) 8.9° ± 6.6°, *p* = 0.001). The pain in both sides was significantly reduced six months after surgery (rest: 2.2 ± 0.9 vs. 0.6 ± 1.5; movement: 7.3 ± 1.1 vs. 1.6 ± 2.4; for both *p* = 0.001) but no sex difference could be found. The gait pattern and stair climbing ability were better in men (*p* = 0.008; *p* = 0.037), but no significant differences in postoperative satisfaction or quality of life could be detected (*p* = 0.671; *p* = 0.409). The combination of a stem with a low offset and cups larger than 55 mm showed better results in the Harris Hip Score (HHS) (mean: 86.9 ± 10.7 vs. 95.3 ± 4.0; *p* = 0.001), what can be considered an advantage for men.

**Conclusions:**

This study shows sex differences in the range of motion and mobility in everyday life. Although the female sex showed better mobility, no differences in satisfaction or quality of life were found six months after surgery. However, the combination of stem and cup size seems to have a relevant influence on postoperative outcome, especially in men.

**Supplementary Information:**

The online version contains supplementary material available at 10.1186/s13018-026-06811-6.

## Introduction

 Total hip arthroplasty (THA) is a well-established procedure for the treatment of end-stage hip osteoarthritis and other debilitating hip conditions. Millions of people are affected by osteoarthritis globally, with significant impairment of their quality of life [1]. The primary goal of the procedure is the alleviation of pain, the restoration of joint function, and the improvement of patients’ quality of life. Over the past decades, advances in prosthesis design, materials, and surgical techniques have led to a substantial rise in the number of procedures. Despite the overall high success rates, a considerable proportion of patients report persistent functional limitations or dissatisfaction following THA, underlining the importance of identifying factors that influence postoperative outcomes [2, 3]. Patient-reported outcome measures (PROMs) are essential for measuring the success of the procedure. Pain, mobility, satisfaction and questions about quality of life are integrated into these studies. Especially the Harris Hip Score is widely used to benchmark outcomes and guide postoperative care and rehabilitation [4]. Several demographic and clinical factors have been proposed to influence the outcomes after total hip arthroplasty. Among these, sex differences have been investigated in numerous studies, with divergent findings. For example, Delanois et al. 2018 revealed that for men the postoperative pain management is important, whereas for women the staff responsiveness is more crucial for a good rating result. Furthermore, other factors like age, body-mass-index (BMI) and comorbidities play a crucial role for the postoperative satisfaction [5, 6]. Some trails suggest high ranges of motion and a better functional recovery for women, while others indicate no clinically relevant differences in satisfaction or complication rates between sexes [7–9]. Additionally, implant factors, such as the size and positioning of the acetabular component and femoral stem, have also been implicated in postoperative success. Some studies propose that larger cup sizes and precise placement within so-called “safe zones” are thought to reduce the risk of dislocation and promote joint stability without negatively influencing satisfaction rates [10]. Other research indicated, that the positioning of the cup regarding the inclination in 44.4 ± 6.94 degrees does not influence the outcome of the patients [11–13]. For loosening Peter et al. described a 4.1% risk for 56 mm cups or above and a risk of 1.3% for cups 54 mm or below [14]. Finally, the comprehensive management of THA patients requires attention to modifiable risk factors, such as sex, preoperative functional status and postoperative rehabilitation. The primary objective of this study was to compare perioperative and short-term post-operative outcomes between male and female patients with advanced osteoarthritis. Specifically, the study aimed to analyse differences in mobility, range of motion and pain, as well a the choice of the implant size between the two sides at six months postoperatively. Additionally, the study sought to investigate the impact of patient sex on postoperative results including functional outcomes, quality of life (QoL), and patient satisfaction. By examining these variables in a prospective cohort, the study intends to provide evidence to guide implant selection and optimize a sex-related clinical decision-making undergoing primary THA.

## Methods

### Study design

This investigation adhered to the STROBE guidelines for reporting observational research in epidemiology. The study protocol respected the principles of the Declaration of Helsinki and received approval from the university hospital’s Ethics Committee [15].

**Fig. 1:**
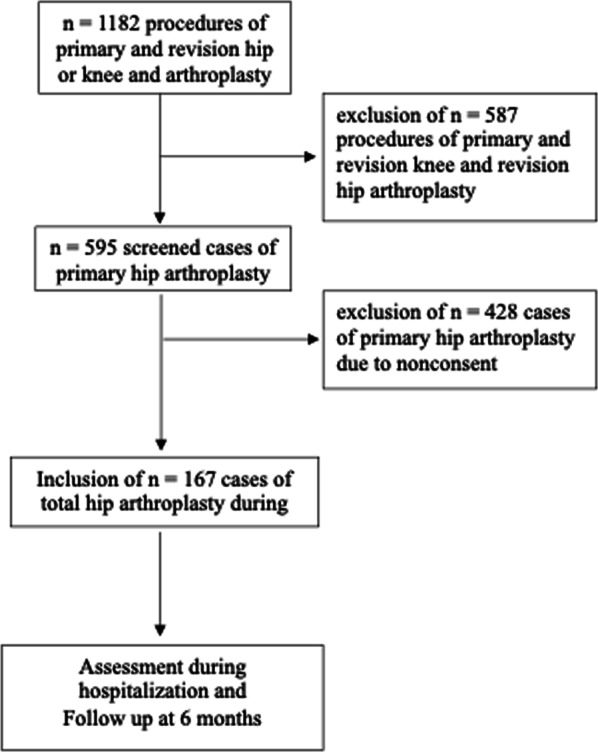
Study design

Patient records were identified for individuals who had primary total hip arthroplasty between June 2022 and June 2023. Data were gathered consecutively during inpatient care and additionally through a six-month prospective follow-up as outlined in Fig. 1. The Pegasos 7 system (Nexus Marabu GmbH, Berlin, Germany) was employed for standard data extraction, with all data organized within Microsoft Excel (Microsoft Corporation, Redmond, WA, USA). nitial sample size calculations anticipated a strong statistical power (80%), with alpha set at 0.05, and effect size of 0.35, focusing on sex as a predictor in the pre-study power analysis. After refining the analysis, fewer variables were investigated, sufficient to ensure robust perioperative and postoperative evaluation with an adequate patient cohort at six months. At admission, demographic and clinical variables were recorded, including: age, biological sex as recorded in the clinical chart, surgical side, BMI, inpatient and intensive care unit duration, pre-existing comorbidities (expressed as the median), and American Society of Anesthesiologists (ASA) classification (ranging 1: healthy, up to 6: brain death) [16]. Throughout hospitalization, data points included type of hip replacement, reported pain before and after surgery, patient mobility and support devices, laboratory results (hemoglobin, CRP, sodium, potassium, INR), occurrences of systemic and surgical complications, and blood transfusion frequency. Systemic events covered respiratory, cardiovascular, urogenital, and neurological domains. Surgical complications comprised early infections, neurological impairment, fractures, bleeding, aseptic loosening, revision procedures, and complications following discharge such as infection or joint instability. The primary outcome parameters were the assessment of the Joint mobility, the Range of Motion and the Harris Hip Score after six months. Secondary parameters were the quality of life, numeric rating scale, quality of life and implant sizes.

### Exclusion criteria

Patients whose records could not be obtained were omitted from the analysis. Individuals who underwent revision hip arthroplasty for either aseptic or septic reasons, as well as those without available or granted consent, were not considered for this study.

### Inclusion criteria

Eligibility encompassed all individuals who received primary total hip arthroplasty. Those included were: (1) patients diagnosed with primary or secondary osteoarthritis; (2) patients aged between 20 and 90 years; (3) cases with full access to clinical data; and (4) those who had provided written informed consent.

### Perioperative management

The total hip arthroplasties were conducted by applying the Zimmer Biomet Fitmore stem and Allofit/Alloclassic cup system. The selection of the prothesis design was the result of many years of using these implants as “in-house standard” at our surgical center with access to long-term results. In addition, comparability was ensured by using a single manufacturer for all implants, and the implants are also used globally. A minimally-invasive anterolateral approach in lateral position was used for all cases. All patients received general anesthesia and at least short-term monitoring after the operation. A stationary or ambulant rehabilitation program was organized prior to hospital release.

### Blood unit supply

The criteria for administering blood transfusions followed the restrictive recommendations outlined by Cochrane: patients with hemoglobin values above 8.0 g/dL did not receive transfusions; those with levels between 7 g/dL and 9 g/dL, combined with clinical symptoms like dizziness, nausea, feeling unwell, or appetite loss, warranted consideration; and values below 8 g/dL definitively indicated the need for transfusion [17].

### Appointment after six months

Enrollment required a signed informed consent information. Patients were examined by the same person, who collected the perioperative data (except ROM). The six-month routine check-ups were carried out by the university health center. The patient was asked to bring all possible data from the rehabilitation center. Furthermore, the patients were asked for their satisfaction (1–5, no satisfaction to completely satisfied), quality of life (QoL) (1–5, significantly poorer QoL to significantly improved QoL), and Harris-Hip-score (1–4, excellent, good, moderate, poor) [18]. Range of motion measurements pre- and postoperatively were performed by the informing surgeon according to standardized clinical goniometry protocols. The six months controls were performed by the doctoral student. All measurements followed established orthopedic practice. The inter-observer reliability for ROM measurements was moderate (ICC = 0.73, 95% CI 0.66–0.79; *p* = 0.001).

### Statistical analyses

All statistical evaluations utilized IBM SPSS version 29.0 (IBM, Armonk, NY, USA) for data processing. Continuous variables were summarized with the mean, standard deviation, and variance. For nominal and dichotomous data, Fisher’s exact test was applied. Independent samples were assessed using T-tests, variance analyses, Levene’s test, and the Welch test. Effect sizes were reported as Cohen’s d (small: 0.20; medium: 0.50; large: 0.80) and the phi coefficient for specific comparisons (small: 0.10; medium: 0.30; large: 0.50), with a 5% interval considered. Correlations between continuous and ordinal variables were examined with Pearson and Spearman methods. Group differences in ordinal and nominal data were analysed with the Mann–Whitney U test and Kruskal–Wallis H test (KWH). For nominal variables with several categories and measurements at two separate time points, repeated measures linear modeling was employed. Differences in pain scores between study groups were determined using multivariate analysis of covariance (MANCOVA), allowing for baseline variation adjustments and more precise group comparisons (see Table 1). Multilinear regression analyses were performed for the key continuous variables total ROM, leg flexion and HHS with sex as a main predictor and age, BMI, ASA and the number of comorbidities as covariates. Total joint mobility was calculated as the sum of patient values for flexion, extension, abduction, adduction, and both internal and external hip rotation. Statistical significance was set for two-tailed tests at α = 0.05.

**Table 1:** Parameters and statistical tests

Parameter	Statistical test	covariates
Mobility	Fischer’s exact test	No
Range of motion (ROM)	Independent samples T-Test	No
Numeric Rating Scale (NRS)	Multivariate Analysis of Covariance	No
Quality of Life (QoL), Harris Hip Score	Fischer’s exact test	No
Implants (I)	Fischers’s exact test	No

## Results

### Recruitment process

In total, data from 167 patients with primary THA and a signed consent information were retrieved from June 2022 to June 2023. Data of all 167 patients were retrieved during hospitalization, and in the six months follow-up.

### Patient demographics

The indications for total hip arthroplasty were 59.9% primary (100 of 167) and 40.1% (67 of 167) secondary hip arthrosis. A total of 21.1% (35 of 167) operations were carried out by resident physicians, 26.3% (44 of 167) were performed by the chief physicians and 52.6% (88 of 167) were performed by senior residents. More demographic data is shown at Table 1.

**Table 2:** Patient demographics; p = significance

Demographics	Men	Women	*p*
Age (years)	66.92 ± 9.22	66.17 ± 11.25	n.s.
sex (%) (*n* = 167)	39.5	60.5	
BMI	28.34 ± 4.20	27.97 ± 5.63	n.s.
ASA Score	1.77 ± 0.79	1.83 ± 0.76	n.s.
Pre-diseases (median)	3.00	3.00	
Primary hip osteoarthritis (*n* = 67) (%)	39.0	61.0	n.s.
Secondary hip osteoarthritis (*n* = 100) (%)	40.3	59.7	n.s.
Proportion of dysplastic coxarthrosis (*n* = 57) (%)	38.6	61.4	n.s.

### Mobility

The preoperative, postoperative, and reappointment walking distance and the use of crushes were not significantly different between male and female. For more information see Tables 2 and 3.

**Table 3:** walking aids preoperatively and after six months

		None	Forearm Crutches	Cane	Walker	Wheelchair	(p); phi
Preoperative	Women (*n* = 101)	63.6%	30.3%	–	6.1%	–	0.059; 0.187
	Men (*n* = 66)	80.3%	18.2%	–	1.5%	–	
6 months	Women (*n* = 101)	71.2%	4.0%	23.8%	–	1.0%	0.602; 0.162
	Men (*n* = 66)	81.8%	1.6%	16.6%	–	–	

**Table 4:** walking distance preoperatively and after six months;

		Unlimited	Up to 1000 m	Up to 500 m	Up to 50 m	(p); phi
Preoperative	Women (*n* = 101)	8.2%	–	54.0%	37.8%	0.491; 0.093
	Men (*n* = 66)	9.1%	–	62.1%	28.8%	
6 months	Women (*n* = 101)	53.5%	29.7%	15.8%	1.0%	0.293; 0.142
	Men (*n* = 66)	66.7%	22.7%	9.1%	1.5%	

### Range of motion

For the range of motion hip flexion, abduction, adduction, external and internal rotation were collected, as well as the deficits. Pre- and postoperative Extension after 6 months for all patients was zero. For more information see Table 4.

**Table 5:** Range of motion;

Preoperative	Men (*n* = 66)	Women (*n* = 101)	(*p*), 95% CI
Flexion	83.72 ± 12.30	83.32 ± 13.47	0.853, [-3.680/4.451]
Abduction	11.80 ± 5.52	13.41 ± 6.16	0.083, [-3.447/0.251]
Adduction	8.86 ± 4.00	9.80 ± 4.84	0.192, [-2.354/0.4773]
External rotation	11.21 ± 5.95	10.90 ± 6.68	0.752, [-1.681/2.323]
Internal rotation	4.77 ± 4.24	6.48 ± 5.22	**0.021**,** [−3.168/-0.256]**
Flexion deficits	46.28 ± 12.52	46.67 ± 13.47	0.852, [-4.451/3.680]
Abduction deficits	28.18 ± 5.52	26.58 ± 6.16	0.083, [-0.211/3.406]
Adduction deficits	28.18 ± 5.52	26.58 ± 6.16	0.083, [-0.211/3.406]
Internal rotation deficits	35.22 ± 4.24	35.51 ± 5.22	**0.021**,** [0.256/3.168]**
External rotation deficits	38.78 ± 5.95	39.10 ± 6.68	0.752, [-2.323/1.681]
After 6 Months			
Flexion	98.18 ± 7.42	102.08 ± 7.52	**0.001**,** [−6.237/-1.558]**
Abduction	31.21 ± 5.40	33.56 ± 6.10	**0.010**,** [−4.132/-0.572]**
Adduction	19.85 ± 6.20	21.88 ± 6.11	**0.038**,** [−3.955/-0.111]**
External rotation	26.59 ± 8.73	28.61 ± 7.17	0.128, [-4.565/0.519]
Internal rotation	8.86 ± 6.60	13.27 ± 6.83	**0.001**,** [−6.512/-2.295]**
Flexion deficits	31.1 ± 7.40	27.92 ± 7.52	**0.001**,** [1.558/6.236]**
Abduction deficits	8.78 ± 5.40	6.43 ± 6.09	**0.010 [0.572/4.132]**
Adduction deficits	10.15 ± 6.20	8.10 ± 6.10	**0.038**,** [0.110/3.954]**
Internal rotation deficits	31.13 ± 6.60	26.73 ± 6.83	**0.001**,** [2.295/6.512]**
External rotation deficits	3.40 ± 8.73	1.38 ± 7.71	0.118, [−0.518/4.564]

### Clinical data

There was no significant sex difference for the BMI, the ASA-score, the number of diseases, the time of surgery, surgical and systemic complications or the number of unplanned follow-up-surgeries (see Table 5).

**Table 6:** Clinical data; MANCOVA analyses; partη^2^ = partielles Eta-Quadrat; n. a. = not available; n. s. = not significant

	Men (*n* = 66)	Women (*n* = 101)	(*p*), partη^2^
BMI	28.54 ± 4.23	27.85 ± 5.43	0.399, 0.005
ASA	1.77 ± 0.79	1.83 ± 0.76	0.577, n. a.
Number of diseases (median)	3.00	3.00	n. s.
Time of surgery	55.52 ± 20.42	54.38 ± 17.60	0.713, 0.001
Surgical complications	0.05 ± 0.20	0.05 ± 0.25	0.895, 0.000
Systemic complications	0.06 ± 0.25	0.07 ± 0.264	0.814, 0.000
Number of unplanned follow up surgeries	0.03 ± 0.178	0.01 ± 0.103	0.339, 0.006

### NRS (numeric rating scale)

The preoperative and postoperative NRS for pain from 1 to 10 after six months did not show any significant sex differences (see Table 6). Overall, the pain in rest and movement was significantly reduced postoperative after six months (rest: 2.22 ± 0.86 vs. 0.59 ± 1.54; movement: 7.34 ± 1.12 vs. 1.62 ± 2.39; for both *p* = 0.001).

**Table 7:** NRS (numeric rating scale (1–10)) pain; MANCOVA analyses;

Preoperative	Men (*n* = 66)	Women (*n* = 101)	(*p*), part η^2^
NRS in rest	2.08 ± 0.70	2.40 ± 1.00	0.091, 0.031
NRS in movement	7.42 ± 0.84	7.30 ± 1.27	0.588, 0.003
postoperative			
NRS in rest	0.58 ± 1.66	0.70 ± 1.80	0.763, 0.001
NRS in movement	2.08 ± 2.50	1.57 ± 2.30	0.318, 0.011

### Quality of life

Regarding the everyday life after six months sex differences could be shown for the total mobility of the replaced hip joint, climbing stairs and during walking (normal or limping). For the use of walking aids (*p* = 0.602, phi = 0.162) and pain medication (*p* = 0.538, phi = 0.110), sitting (30 min: m 12.1% vs. w 12.9%; 60 min m 87.9% vs. w 87.1%; *p* = 1.0), continuing physiotherapy after rehabilitation, satisfaction with the surgery and the quality of life and the Harris Hip Score (HHS) no significant differences between female and male patients were found (see Table 7).

**Table 8:** Postoperative everyday life; ROM = range of motion

Total ROM (U-Test: middle ranks)	Men (*n* = 66)	Women (*n* = 101)	(*p*), phi
	70.83	92.61	0.001, (Z) -3.195
Climbing stairs			
Challenging	12.1%	27.7%	
Natural with the use of handrails	27.3%	34.7%	**0.008**,** 0.241**
Natural without the use of handrails	60.6%	37.6%	
Gait pattern			
Significant limping	6.1%	6.9%	
Slight limping	24.2%	42.6%	**0.037**,** 0.195**
Natural	69.7%	50.5%	
Physiotherapy after institutional rehabilitation			
None	9.1%	6.9%	
Regulary	86.4%	89.1%	0.870, 0.043
Irregulary	4.5%	4.0%	
Satisfaction with surgery			
Highly satisfied	84.8%	85.1%	
Partially satisfied	7.6%	9.9%	0.671, 0.065
Not satisfied	7.6%	5.0%	
Quality of life			
High	68.2%	64.4%	
Average	24.2%	31.7%	0.409, 0.105
Low	7.6%	4.0%	
Harris hip score			
Excellent	53.0%	51.5%	
Good	21.2%	17.8%	
Moderate	12.1%	18.8%	0.678, 0.094
Poor	13.6%	11.9%	

When looking at the male and female sexes separately, it became apparent that, despite normal gait patterns and stair climbing ability, the quality of life reported by both groups was lower than their level of satisfaction. For example, 83.3% of the men (*n* = 55) with normal to slightly limping gait were highly satisfied, but only 68.2% (*n* = 45) described a high postoperative quality of life (*p* = 0.007, phi = 0.484; *p* = 0.001, phi = 0.583). Similar results were shown for stair climbing with 77.3% (*n* = 51) highly satisfied men who could climb stairs normally with or without handrail, but only 68.2% (*n* = 45) described a high quality of life (*p* = 0.001, phi = 0.541; *p* = 0.001, phi = 0.641).

78.2% of the women (*n* = 79) with normal to slightly limping gait were highly satisfied, but only 64.5% (*n* = 65) described a high postoperative quality of life (*p* = 0.007, phi = 0.484; *p* = 0.001, phi = 0.500). Similar results were shown for stair climbing with 85.1% (*n* = 86) highly satisfied men who could climb stairs normally with or without handrail, but only 57.4% (*n* = 58) described a high quality of life. Additionally, 18.8% (*n* = 19) of the female patients with an impaired stair climbing were still highly satisfied, but only 6.9% described a high quality of life (*p* = 0.005, phi = 0.343; *p* = 0.001, phi = 0.541).

### Multilinear regression analyses

For the total range of motion sex could be shown as the main predictor of significant differences. Women showed a 13.93° greater range of motion than men (B = 13.93, β = 0.253, *p* < 0.001). Higher BMI demonstrated a significant negative association with ROM (B = -1.18° per kg/m^2^, β = -0.223, *p* = 0.004), equivalent to approximately 6° ROM reduction per 5-unit BMI increase (e.g., normal to obese range). Age, ASA score and comorbidities showed no confounding effect. Again, for leg flexion sex revealed to be the main predictor for significant differences. Female sex remained the strongest independent predictor (B = 3.77°, β = 0.240, *p* = 0.001), with women exhibiting 3.77° greater flexion compared to men after controlling for age, BMI, ASA score, and comorbidities. Higher BMI showed a significant negative association (B = −0.48° per kg/m^2^, β = −0.316, *p* = 0.001), translating to 2.4° flexion loss per 5-unit BMI increase – the second strongest predictor. Comorbidities were significantly detrimental (B = -0.97° per comorbidity, β = −0.302, *p* = 0.044). For the HHS sex and the covariates (Age, ASA score, BMI and number of comorbidities) were not predictors for the outcome.

### Implants

Regarding the implants, no significant sex difference was found in the use of the stem design only (Fitmore A or B). In combination with the use of cup sizes separated into two groups ≤ 54 mm versus ≥ 56 mm), significant sex differences could be revealed when considering stem design A or B (see Table 8). Regarding HHS, no sex differences were found in the Bonferroni post hoc analyses (see Table 9). Significant differences in the results could be found between Fitmore A plus ≤ 54 mm and ≥ 56 mm cups, with better results for the A plus ≥ 56 mm group (mean: 86.87 ± 10.74 vs. 95.25 ± 4.00; *p* = 0.001). For Fitmore B plus ≤ 54 mm and ≥ 56 mm cups no significant difference for the HHS was shown (mean 84.89 ± 13.38 vs. 84.30 ± 17.06; *p* = 0.859). For the quality of life and the satisfaction after surgery no significant differences could be shown for the Fitmore A and B groups with ≤ 54 mm and ≥ 56 mm cups (*p* = 0.787; *p* = 1.0; *p* = 0.312; *p* = 0.346) (Table 10).

**Table 9:** implant size

	Men (*n* = 66)	Women (*n* = 101)	(*p*), phi
Fitmore A Fitmore B	*n* = 19 *n* = 47	*n* = 34 *n* = 67	0.610, -0,051
Fitmore A + Cup ≤ 54 mm Fitmore A + Cup ≥ 56 mm	*n* = 6 *n* = 10	*n* = 26 *n* = 5	**0.001**,** − 0.533**
Fitmore B + Cup ≤ 54 mm Fitmore B + Cup ≥ 56 mm	*n* = 27 *n* = 23	*n* = 66 *n* = 4	**0.001**,** − 0.513**

**Table 10:** Harris Hip Score and sex analyses with implant combinations; HHS = Harris Hip Score

	Men (*n* = 66)	Women (*n* = 101)	HHS	(p)
Fitmore A + Cup ≤ 54 mm	*n* = 4	*n* = 26	80.85 vs. 87.85	0.727
Fitmore A + Cup ≥ 56 mm	*n* = 9	*n* = 4	95.35 vs. 96.00	
Fitmore B + Cup ≤ 54 mm	*n* = 26	*n* = 65	86.85 vs. 84.02	
Fitmore B + Cup ≥ 56 mm	*n* = 20	*n* = 2	82.95 vs. 90.50	

## Discussion

In this prospective study of 167 patients undergoing total hip arthroplasty (THA), which examined sex differences, we found that sex differences occurred for the range of motion, mobility, climbing stairs, the gait pattern after six months and the design and size of implants used for the arthroplasties. Specifically, men were less able to internally rotate, flex, abduct and adduct and, as a logical consequence, had poorer overall joint mobility. Interestingly, regarding stair climbing and gait pattern, men were significantly better than women. As expected, the cup sizes of men were highly significantly larger than for women, but no significant sex difference was found in selecting stem design A or B. Despite women show a better range of motion and mobility and requiring smaller cup sizes, no significant sex difference for quality of life, satisfaction or the Harris Hip Score could be found after six months of surgery. These are interesting findings, as the range of motion and mobility are major criteria’s for defining a good clinical outcome. For example, Ackerman et al. (2024) revealed that dissatisfaction and patient-perceived worsening after THA were associated with a higher likelihood of revision (relative risk (RR) 10.18, 95%CI 6.01–17.25) [19]. Muscular strength and a good range of motion lead to success and pain reduction in the early postoperative rehabilitation. Improvement of hip function after THA is also achieved when postoperative physiotherapy targets the strength training of hip muscles within the first postoperative week [20, 21]. Also, postoperative quality of life and patient satisfaction show improvement from approximately six weeks after surgery [22]. In this study, the postoperative quality of life and satisfaction did not show any sex differences with high satisfaction rates above 80% and above 60% quality of life in both groups (*p* = 0.671; *p* = 0.409). In half of the patients in both groups an excellent Harris Hip Score was found (*p* = 0.678). Interestingly, the total range of motion was highly significantly different between male and female patients (*p* = 0.001) in favor of the women, whereas the male patients demonstrated a better gait pattern and stair climbing capacity (*p* = 0.037; *p* = 0.008). In this study ROM and flexion advantage in women remained statistically robust, indicating a primarily sex-specific effect rather than confounding by BMI or comorbidities. Nevertheless, BMI represents a modifiable factor influencing postoperative mobility outcomes. It would be expected that these results have an impact on satisfaction and thereby lead to sex-specific differences in the outcome. A differentiated closer look onto gait pattern and stair climbing for male and female patients revealed that the satisfaction with the postoperative result was higher than the quality of life in both groups. Furthermore 18.81% of the female patients with highly impaired ability to climb stairs were still highly satisfied with the surgery, but less than 7% described an excellent quality of life. However, since the results of this study showed no difference, the question arises as to which factors other than range of motion and mobility in everyday life have a significant influence on satisfaction. For example, Delanois et al. 2018 revealed that pain management and reduction was a more important factor for men than women with regard to postoperative satisfaction (*p* = 0.021) [6]. In addition, preoperative patient expectations may play a role in postoperative satisfaction [23]. Neuprez et al. 2016 could show in multivariate analyses, that higher expectation scores predicted higher satisfaction after THA (*p* = 0.001) [24].

In this study, postoperative pain relief, walking distance and the use of walking aids did not show a significant sex difference. These findings are supported by the existing literature [25–27]. Yet, the postoperative pain relief in both groups was significant (*p* = 0.001). Halawi et al. 2019 published that 41% of the THA patients in their study were dissatisfied when pain persisted [28].

To analyse weather implant sizes could influence the postoperative HHS for the stem types two groups were built with the paired cups above and below 54 mm. As expected for Fitmore A and B the implantations with cups from 56 mm were more likely to be implanted for male patients (both *p* = 0.001). Intriguingly, patients with Fitmore A and cup sizes from 56 mm showed a higher HHS (mean: 86.87 ± 10.74 vs. 95.25 ± 4.00; *p* = 0.001). As male patients were more likely to get implanted Fitmore A stems with cup sizes from 56 mm an above, these results could be interpreted as a sex difference in favor for male patients. No concrete evidence of a correlation between implant design and improved satisfaction and mobility could be found in the current literature, although personalized implants or 3D-printed implants and the implantation of the cup into the “Lewinnek safe zone” were important factors [29–32]. Further biomechanic studies could possibly provide a mechanical explanation of the significant results in this study.

While our findings suggest advantages for both sex groups, with a better range of motion for female patients better results for men regarding gait pattern and stair climbing, there are several limitations that warrant caution in interpretation. Our single-center design, while ensuring procedural consistency, limits extrapolation to institutions using different implant systems or rehabilitation protocols. Furthermore, the non-randomized allocation may have introduced a potential selection bias, as surgeons might have preferentially selected Fitmore A or B implants for the patients. The 6-month follow-up of this study can provide early insights into the postoperative outcomes and initial implant function but does not capture potential late complications, implant failures, or the true longevity of the devices. Durability assessments would require longer-term data to evaluate whether implants maintain their integrity, function, and safety over time. Therefore, at least 1–5 years of follow-up will be necessary to observe whether the early benefits persist or if issues such as wear, loosening, or delayed adverse events might emerge. In addition, the limited sample size may also influence the results of this investigation. Future studies should also employ standardized goniometry protocols with a strict inter-observer reliability to enhance measurement consistency.

Surgeons should consider that both sex substantial improve in pain, mobility and daily function after six months. Although the range of motion and mobility deficits differ between males and females no influence on the quality of life or satisfaction after six months could be shown. Researchers are encouraged to conduct larger, multicenter, and long-term studies to validate these findings. Future research should also investigate why female and male patients report a lower quality of life despite good satisfaction and aim to identify strategies to optimize patient-reported outcomes individualized for both sexes.

In this analyse of sex-specific outcomes both sexes experienced substantial improvements in pain and function at six months postoperatively, with no significant differences in patient satisfaction, quality of life, or complication rates. Women demonstrated advantages in the total range of motion and leg felxion with less motion deficits, especially for internal rotation, whereas men showed a better gait pattern and stair climbing. Both sexes benefited from surgery with regard to pain relief and most of them were highly satisfied with the surgery. Interestingly, the postoperative quality of life was less pronounced than satisfaction without any clear indication of a specific reason. The selected implants could provide an indication, with significantly improved HHS results for stems with lower offset and larger cup components. Further large-scale, long-term randomized studies are warranted to clarify the optimal implant combination for both sexes.

## Supplementary Information

Below is the link to the electronic supplementary material.

Supplementary Material 1

## Data Availability

The datasets used and analysed during the current study are available from the corresponding author on reasonable request.

## Abbreviations

ASA: The American Society of Anesthesiology
BMI: Body mass index
CRP: C reactive protein
CR: Cruciate retaining
Hb: Hemoglobin
HHS: Harris Hip Score
KWH: Kruskal–Wallis H
η2p: Partial eta square
PS: Posterior stabilized
PROMs: Patient reported outcome measures
QoL: Quality of life
ROM: Range of motion
THA: Total hip arthroplasty
