# Supplementary material for: Comparative analysis of postoperative outcomes in men and women following total hip replacement surgery
Source: J Orthop Surg Res. 2026 Apr 3;21:248. doi: 10.1186/s13018-026-06811-6 (PMC13064225; doi:10.1186/s13018-026-06811-6)
Supplement: Supplementary file 1 — Supplementary Material 1 [file 13018_2026_6811_MOESM1_ESM.pdf]

STROBE Item	Manuscript Details
**Title and Abstract (Items 1a, 1b)**	Study design is described as prospective cohort in title and abstract. Abstract provides balanced summary of objectives, methods, results, and conclusions.
**Background/Rationale (Item 2)**	Scientific background and rationale regarding gender differences in total hip arthroplasty (THA) clearly explained.
**Objectives (Item 3)**	Specific aims to investigate perioperative and postoperative gender differences are clearly stated.
**Study Design (Item 4)**	Prospective cohort design described early in methods section, referencing STROBE adherence.
**Setting (Item 5)**	Conducted at a university hospital; recruitment from June 2022 to June 2023 with detailed data collection periods.
**Participants (Items 6a, 6b)**	Inclusion and exclusion criteria clearly defined; 167 patients enrolled with informed consent; no matched controls.
**Variables (Item 7)**	Outcomes (range of motion, pain, satisfaction), exposures (gender, implant size), predictors, and confounders detailed.
**Data Sources/Measurement (Item 8)**	Data extracted from patient records and follow-up visits; measurement tools and software (SPSS, Pegasos 7) described.
**Bias (Item 9)**	Potential biases acknowledged in discussion (single-center, nonrandomized implant allocation); addressed limitations.
**Study Size (Item 10)**	Sample size calculated a priori with power analysis; 167 patients included, adequate for planned analyses.
**Quantitative Variables (Item 11)**	Quantitative variables like range of motion and pain scores thoroughly analyzed; grouping and handling explained.
**Statistical Methods (Items 12a-e)**	Detailed statistical analyses including t-tests, MANCOVA, Mann-Whitney U, effect sizes, confounder adjustment, subgroups described; missing data and loss to follow-up addressed.
**Participants (Items 13a-c)**	Flow of participants through study stages clear; inclusion, follow-up, and analysis numbers provided; non-participation reasons discussed. Flowchart suggested.
**Descriptive Data (Items 14a-c)**	Demographics and baseline data summarized in tables; missing data reported; follow-up time summarized.
**Outcome Data (Items 15a-c)**	Outcome events and summary measures (range of motion, satisfaction, etc.) reported with confidence intervals where applicable.
**Main Results (Items 16a-c)**	Key results about gender differences and implant effects clearly presented with appropriate statistical data.
**Other Analyses (Item 17)**	Subgroup analyses (implant size effects) and interactions reported with sensitivity analyses considerations.
**Key Results (Item 18)**	Results summarized with respect to study objectives in discussion section.
**Limitations (Item 19)**	Limitations (single-center, nonrandomization, follow-up duration) openly discussed with potential bias impact.
**Interpretation (Item 20)**	Balanced interpretation of findings with consideration of limitations and comparison to existing literature.
**Generalizability (Item 21)**	External validity discussed; limitations due to single center and specific implant systems noted.
**Funding (Item 22)**	No funding or sponsor influence declared.
